# Barriers to Care for Newly Diagnosed HIV Patients: Insights From a Single-Centre Study

**DOI:** 10.1155/arat/7548833

**Published:** 2025-03-29

**Authors:** Jo Yen Yong, Nor Zaila Zaidan, Wee Fu Gan

**Affiliations:** Infectious Diseases Unit, Melaka General Hospital, Melaka, Malaysia

**Keywords:** CD4, healthcare system barrier, HIV, individual barrier, late presenter, opportunistic infection

## Abstract

**Introduction:** We aimed to evaluate the clinical presentation and diagnosis process of all newly diagnosed human immunodeficiency virus (HIV) patients and conduct a ‘look back' for barriers to care to aid a greater understanding of interventions to reduce late presentation.

**Methods:** We evaluated 102 patients with newly diagnosed HIV who were referred to Melaka Hospital's infectious disease (ID) team from January 2021 to December 2022 via retrospective case record review. They were categorised into late presenters (LPs) and nonlate presenters (NLPs). LP is defined as persons presenting for care with a cluster of differentiation 4 (CD4) count below 350 cells/μL or presenting with an acquired immunodeficiency syndrome (AIDS) defining event, regardless of the CD4 cell count. Demographic characteristics, individual and healthcare system barriers and treatment outcomes were evaluated.

**Results:** There were 89.2% of LPs, with 56.9% presenting with opportunistic infection (OI). Median CD4 for LPs upon diagnosis was 53 cells/μL. *Pneumocystis jirovecii* pneumonia was the most common presenting OI. Most were men who had sex with men (MSM) with more university graduates among the NLPs compared to LPs (36.4% vs. 8.8%, *p* 0.02). 9.9% of LPs experienced pitfalls during healthcare consultation, leading to late presentation, which was labelled as adverse events. LP's median time from diagnosis to first ID consultation was 7 days, and all patients' median duration of antiretroviral therapy (ART) initiation was 24 days. 82.4% of the patients were still on follow-up, with 69.6% achieving virological suppression at 6 months of ART. The mortality rate was 5.9%, all of which were LPs, and most were MSM.

**Conclusions:** Late presentation remains challenging, with 9.9% of potent preventable adverse events. Morbidity meetings are crucial for immediate feedback to involved healthcare providers. Community-based organisations also play an essential role in identifying and providing linkage of high-risk groups to early HIV screening and diagnosis.

## 1. Introduction

With the advances in human immunodeficiency virus (HIV) treatment in this era, a renewed vision to reach 95-95-95 testing and treatment targets by 2025 was introduced by the Joint United Nations Programme on HIV/AIDS (UNAIDS) [1].

However, late presentation among newly diagnosed HIV patients remains a global challenge, contributing to higher morbidity and mortality rates [2]. Understanding barriers to care is critical for timely interventions.

Late presenter (LP) is defined as a person presenting for care with a cluster of differentiation 4 (CD4) count below 350 cells/μL or presenting with an acquired immunodeficiency syndrome (AIDS) defining event, regardless of the CD4 cell count [3]. Late entry to HIV care appears to have higher mortality rates, and recruiting HIV-infected individuals to care earlier could lead to substantial improvements in antiretroviral therapy (ART) outcomes [4].

This is also supported by the INSIGHT trial, which demonstrated that early ART initiation was associated with beneficial effects for both serious AIDS-related and non-AIDS-related events, compared to patients who received ART after CD4 cell count declined to less than 350 cells/μL [5].

A local study reported a high proportion of LPs among newly diagnosed HIV patients, indicating that late presentation for testing may be pervasive [6]. A higher proportion of expenditure was also being spent on AIDS care and treatment rather than prevention, according to local HIV data [7].

The study's primary purpose is to review the clinical presentation and diagnosis process of all newly diagnosed HIV patients at Melaka Hospital between January 2021 and December 2022. This will aid in identifying both individual and healthcare system barriers and epidemiological risk factors that contribute to late diagnosis or presentation. The outcomes of all the recruited patients were also studied. These findings are expected to yield significant insight and guide the implementation of interventions to improve the system and delivery of HIV care.

## 2. Materials and Methods

### 2.1. Study Setting and Population

This retrospective case record review was conducted at the Melaka Hospital, a tertiary care facility in Melaka, Malaysia. This hospital is a primary regional healthcare provider, offering various medical services, including specialised care for infectious diseases (IDs).

The study focussed on all ART naïve newly diagnosed HIV patients who presented to the Melaka Hospital ID team between January 2021 and December 2022. During this period, the team received approximately 108 HIV diagnoses. Six patients were excluded as they were known to have HIV and had a history of exposure to ART before presentation. Patients were categorised as LPs or nonlate presenters (NLPs). LP was defined as a person presenting for care with a CD4 count below 350 cells/μL or presenting with an AIDS-defining event, regardless of the CD4 cell count.

Data for this study were extracted from the hospital's HIV registry, which includes detailed information on patient demographics, clinical characteristics, laboratory results and treatment outcomes. The Medical Research and Ethics Committee (MREC), Ministry of Health Malaysia (MOH), approved the study, and all patients' data were anonymised to protect confidentiality.

### 2.2. Data Collection and Definitions

Demographic characteristics such as age, gender, transmission mode, marital status and occupation were collected. Clinical characteristics such as CD4 were recorded upon presentation, and opportunistic infection (OI) was also recorded.

Patients were assessed for individual barriers in pursuit of HIV care, such as perceived healthy status, lack of knowledge on HIV testing location, not knowing about HIV, financial constraints to access healthcare or stigma associated with receiving HIV care.

The presence of healthcare system barriers was also recorded, such as geographic barriers to healthcare facilities, specialist shortages at primary care facilities, late referrals to treatment centres, incorrect clinical judgement by the first attending doctor leading to late diagnosis/missed opportunities and lack of referral tracking from the initial care provider. We define late referrals as patients who were not referred for ID consultation after being diagnosed with HIV for more than 28 days.

To further explore healthcare system barriers, we captured the first healthcare facility that diagnosed patient's HIV, the time from diagnosis to first ID consultation and time to ART initiation and the context of screening (incidental screening, voluntary screening, medical problem, premarital screening, HIV-positive partner, prisoner/refugee screening and pregnancy) for patients who did not present with OI.

Among the patients who presented with OI, if their late presentations were due to healthcare system barriers, the case would be labelled as an adverse event.

Treatment outcome was determined by capturing baseline viral load, date of ART initiation and viral load suppression at 6 months post-ART. Their clinical outcome at 6 months of ART (ongoing follow-up, loss to follow-up and death) was also recorded. Viral load suppression is defined by a viral load of < 20 copies/mL using Alinity M HIV-1 assay, and any value above 20 copies/mL is deemed unsuppressed viral load. We reviewed our patients' data up to 6 months after ART initiation. Hence, loss to follow-up is defined as patients who did not return or were unreachable at the point of follow-up 6 months post-ART initiation.

### 2.3. Statistical Analysis

Statistical analyses were performed using IBM SPSS Statistics (Version 27.0). When not normally distributed, continuous variables were presented as mean and standard deviation (SD) or as the median and interquartile range (IQR). A chi-square test was used to analyse the difference between groups for categorical variables, and Student's *t*-test (or Mann–Whitney *U* test when data were not normally distributed) was used for continuous variables. Statistical significance was defined as a two-tailed *p* value < 0.05.

## 3. Results

From January 2021 to December 2022, 102 patients were diagnosed with HIV infection, and the majority (89.2%) were LPs (Table 1). The median age of LPs was 34 years old, slightly younger than NLPs (median age: 36.64 years old). Most patients from both groups were single, male and of Malay ethnicity. Men who have sex with men (MSM) accounted for the majority of LPs. University graduates were significantly more common among NLPs (36.4% vs. 8.8%, *p* 0.02).  Table 2 further describes the clinical characteristics of recruited patients—58 LPs (56.9%) presented with OI upon diagnosis. Median CD4 for LPs upon diagnosis was 53 and 542 cells/μL among the NLPs. The median time from diagnosis to first ID consultation among LPs was 7 days, compared to 21 days among the NLPs. *Pneumocystis jirovecii* pneumonia (PCP) was the most common OI upon presentation, followed by extrapulmonary tuberculosis (TB) and cerebral toxoplasmosis.

Both individual and healthcare system barriers to HIV care were assessed among the recruited patients (Table 3). 65.9% of the LPs perceived themselves to be healthy, while 20.9% of them presented late due to the stigma associated with HIV care. 7.7% of them did not know about HIV, which led to a delay in presentation and diagnosis. As for the healthcare system barrier, nine (9.9%) LPs experienced pitfalls during their consultation. Four were referred late to the treatment centre, and four were referred, but the patients did not turn up, and there was a lack of referral tracking from the initial healthcare provider. One was misdiagnosed with other medical conditions, leading to a delay in HIV diagnosis. A similar issue also arose among the NLPs; two did not turn up for their appointment with an ID specialist, and the referring clinician lacked referral tracking. An LP with a delayed diagnosis due to a healthcare system barrier will be labelled as an adverse event.

**Figure 1:**
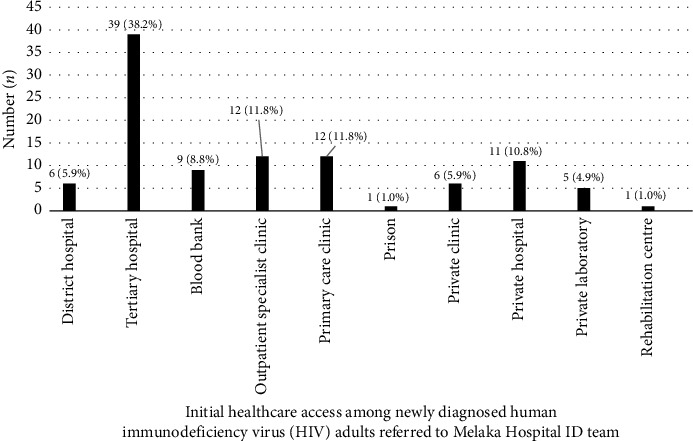
Distribution of initial healthcare access among newly diagnosed human immunodeficiency virus (HIV) adults referred to Melaka Hospital ID team, 2021-2022.

**Figure 2:**
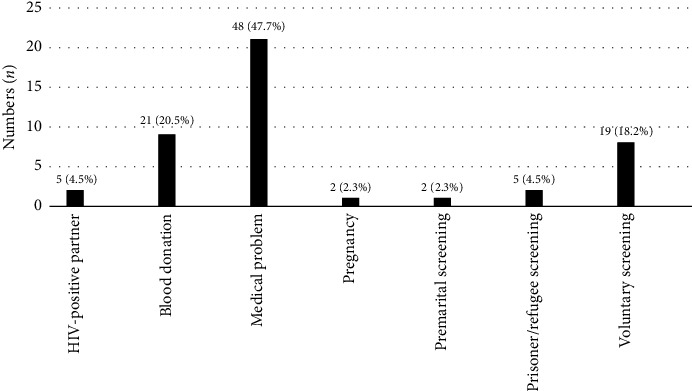
Context of screening for patients without opportunistic infection (OI).

Although most patients were diagnosed in an inpatient setting when they presented with OI, 29.5% were diagnosed in outpatient departments such as specialist clinics and private or primary care clinics (Figure 1). Among the patients who did not present with OI, twenty-one (47.7%) of them were diagnosed with HIV when they presented with other medical problems (such as acute febrile illness, skin problems, visual problems and heart failure), whereas nine (20.5%) of the patients were diagnosed during blood donation, and eight (18.2%) during medical check-up (Figure 2).

**Figure 3:**
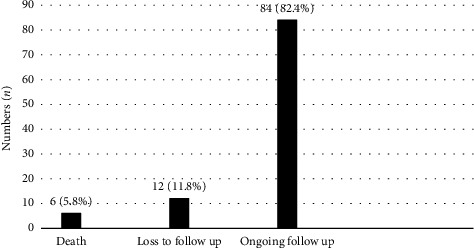
Treatment retention at six months of ART initiation.

**Figure 4:**
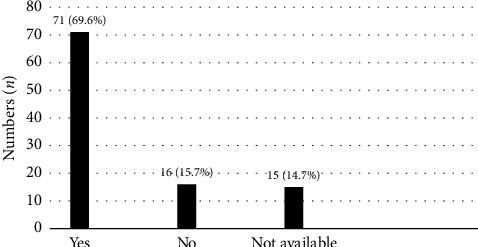
Viral load (VL) suppression at six months of ART initiation.

The median duration from diagnosis to ART initiation among all the patients was 24 days. 82.4% of the patients were still on regular follow-up at 6 months of ART, with 11.8% of them who were lost to follow-up and 5.8% of them died at 6 months of ART initiation (Figure 3). Among patients under our care, 69.6% achieved virological suppression at 6 months of ART, 15.7% did not and 14.7% did not have a repeated viral load (test was not ordered or sample was rejected) (Figure 4).

There were a total of six deaths, and all were LPs. There was no significant difference in their demographic background except for the transmission mode. 50% of them who died were MSM (*p* < 0.001). The median CD4 was 40 cells/μL upon presentation. Four of them had their first ID consultation within 4 days as they were being admitted as inpatients for OI treatment. Another two of them were diagnosed in district or private hospitals, and they were only being referred after 28 days from diagnosis. All of them succumbed to death due to the progression of OI.

## 4. Discussion

Although we have achieved remarkable progress in the treatment and prognosis of patients diagnosed with HIV infection, our study highlights a high prevalence (89.2%) of late presentation among newly diagnosed HIV patients, with OIs and low CD4 counts being common. This has far exceeded the prevalence of LPs in European countries (49%) based on their HIV/AID surveillance data in 2017 [8], and it has also drawn a distance from the 2025 UNAIDS target of 95%-95%-95% [9].

Understanding and identifying the risk factors associated with late presentation is crucial. As opposed to a retrospective cohort study in Greece, which reported that NLPs were majority MSM as they were more aware of their risk behaviour, which contributed to earlier screening and diagnosis, 48.4% of our LPs were MSM [10]. There was also a significant difference in education background between LPs and NLPs (8.8% vs. 36.4%, *p* 0.02), whereby there were more university graduates among the NLPs, suggesting that higher levels of knowledge and awareness have contributed to an earlier diagnosis of HIV. Upon assessing individual barriers leading to late HIV care linkage, many patients perceived themselves as a healthy person with a low risk of HIV infection and did not undergo routine voluntary testing; instead, half of them were diagnosed when they presented with OI. This could be due to a lack of HIV-related knowledge, leading to poor awareness regarding their own infection risk. Increasing public health awareness through targeted campaigns will aid in dispelling myths and misconceptions about HIV. These campaigns should focus on normalising HIV testing and treatment, emphasising the importance of early diagnosis and showcasing testimonials from people living with HIV. In addition, perceived lack of confidentiality and potential discriminatory behaviour at public health facilities were significant deterrents to testing, according to a local study [6]. Hence, getting the high-risk group for voluntary screening is difficult. Community-based organisations become extremely important to reach out to the targeted population to spread awareness and facilitate them with self-testing. Meanwhile, our health ministry has also launched a website which provides HIV risk assessment tools and distribution of self-test toolkits upon request to maintain the individual's confidentiality and, at the same time, increase the numbers of self-testing among the high-risk group [11]. Healthcare providers should also be trained to handle HIV cases with sensitivity and confidentiality. They should be educated on the impact of stigma and discrimination. More effort should also be put into establishing a comprehensive pre-exposure prophylaxis (PrEP) programme for HIV prevention. Providing antiretroviral medication to high-risk individuals significantly reduces the chances of acquiring HIV.

While all the LPs had individual barriers leading to late presentation and diagnosis, we also recorded 9.9% of adverse events, defined by late presentations due to pitfalls in the healthcare system, such as late referral to the treatment centre, lack of referral tracking from the initial care provider and wrong diagnosis due to incorrect clinical judgement leading to late HIV diagnosis. Two (18.2%) NLPs were referred to the treatment centre but did not turn up and were overlooked by the initial healthcare provider. Fortunately, they were not in advanced disease when presented to the treatment centre. Healthcare system pitfalls were also being reported in developed countries whereby even when the patients sought medical attention while experiencing HIV-related symptoms, HIV test was never offered to them [12]. Studies also showed that HIV continued to be considered exceptional by people diagnosed late as well as by their healthcare providers, indicating the high threshold in discussing the topic, both in healthcare and among patients [13]. Immediate feedback to the respective healthcare providers is essential, and revision of workflow and referral system is inevitable to ensure that it is sustainable in the long run and avoid missed opportunities.

Most patients were referred for ID consultation within 7 days of diagnosis. LPs were managed by the ID specialists as early as 3 days, compared to 21 days for NLPs, as most of them were admitted for OIs and inpatient referrals were done. The median duration of ART initiation for all patients, including both LPs and NLPs, was 24 days, primarily influenced by the delays in LPs who presented with OIs. With the improvement of the diagnostic flow, the duration is expected to be shortened as the proportion of LPs will reduce, which also translates to fewer patients with OI. Treatment retention of our patients was as high as 82.4%, with at least 69.6% of them achieving viral load suppression at 6 months of ART initiation. Articles reported that married people were less likely to be lost to follow-up [14, 15]. However, this was not seen in our study as the proportion of single and married patients under our follow-up was similar (58.3% vs. 41.7%). Late presentation with advanced HIV infection was also found to be the significant prognostic factor leading to mortality, as all six deaths occurred among the LPs. An article also reported that single marital status was an independent predictor of death [16]. Although not statistically significant, among our six patients who died, the majority were single (66.7% vs. 33.3%, *p* 0.0694).

## 5. Conclusion

Late presentation remains a significant challenge, with preventable adverse events occurring in nearly 10% of cases. Strengthening healthcare systems through morbidity and mortality reviews, targeted feedback to healthcare providers and robust collaboration with community-based organisations are critical to improving early diagnosis and linkage to care among high-risk populations.

## Figures and Tables

**Table 1:** Demographic characteristics of late presenters (LPs) and nonlate presenters (NLPs).

	LP, *n* (%)	NLP, *n* (%)	*p* value
*N*	91 (89.2%)	11 (10.8%)	
Sex			
Male	81 (89%)	11 (100%)	
Female	10 (11%)	0 (0%)	0.595
Age			
Median age, years old	34	36.64	
Ethnicity			
Chinese	14 (14.7%)	1 (9.1%)	
Malay	72 (79.4%)	9 (81.8%)	
Indian	4 (4.9%)	1 (9.1%)	
Others	1 (1.0%)	0 (0.0%)	0.841
Transmission mode			
MSM	44 (48.4%)	7 (63.6%)	
Heterosexual	28 (30.7%)	2 (18.2%)	
Bisexual	9 (9.9%)	1 (9.1%)	
IVDU	2 (2.2%)	1 (9.1%)	
≥ 2 transmission mode	4 (4.4%)	0 (0.0%)	
Not known	4 (4.4%)	0 (0.0%)	0.830
Education			
Elementary	4 (4.4%)	1 (9.1%)	
High school	51 (56.0%)	2 (18.1%)	
Diploma	28 (30.8%)	4 (36.4%)	
University	8 (8.8%)	4 (36.4%)	**0.022**
Status			
Single	68 (74.7%)	7 (63.6%)	
Married	23 (25.3%)	4 (36.4%)	0.476
Occupation			
Agricultural worker	5 (5.5%)	0 (0.0%)	
Armed force	3 (3.3%)	1 (9.1%)	
Casual sex worker	1 (1.1%)	0 (0.0%)	
Housewife	1 (1.1%)	0 (0.0%)	
Labourer	1 (1.1%)	1 (9.1%)	
Manager	10 (11.0%)	1 (9.1%)	
Professional	10 (11.0%)	3 (27.3%)	
Service and sales worker	39 (42.9%)	3 (27.3%)	
Technician	5 (5.5%)	1 (9.1%)	
Unemployed	16 (17.6%)	1 (9.1%)	0.516

*Note:* MSM, men who have sex with men. Bold value represents the statistically significant *p* value.

Abbreviation: IVDU, intravenous drug user.

**Table 2:** Clinical characteristics of late presenters (LPs) and nonlate presenters (NLPs).

	LP	NLP
Numbers of LPs with opportunistic infection (OI) upon presentation (%)	58 (56.9)	
CD4 (cells/μL) for LPs upon diagnosis		
Median	53	542
Percentile		
25	25	438
75	176	948
Time from diagnosis to first infectious disease (ID) consultation (days)		
Median	7	21
Opportunistic infection (OI) upon presentation		
2014 CDC Stage 1 HIV		7
2014 CDC Stage 2 HIV		4
2014 CDC Stage 3 HIV		
No OI, but CD4 < 200	31	
PCP	25	
Extrapulmonary TB	14	
Cerebral toxoplasmosis	8	
CMV infection	6	
Other OIs	12	
HIV-related		
Cardiomyopathy	1	
HIV retinopathy	1	

*Note:* 2014 CDC case definition for HIV infection according to stage: Stage 1—CD4 count ≥ 500 cells/μL (≥ 26%) with no AIDS-defining condition; Stage 2—CD4 count 200–499 cells/μL (14%–25%) with no AIDS-defining condition; Stage 3—CD4 count < 200 cells/μL (< 14%) or documented of AIDS-defining condition.

Abbreviations: CMV, cytomegalovirus; TB, tuberculosis.

**Table 3:** Individual and healthcare system barriers to human immunodeficiency virus (HIV) care among late presenters (LPs) and nonlate presenters (NLPs).

			LP	NLP
Barriers to HIV care (individual)	Not knowing about HIV	Numbers (*n*)	7	0
		Percentage (%)	7.7%	0.0%
	Financial constraint	Numbers (*n*)	1	0
		Percentage (%)	1.1%	0.0%
	Lack of knowledge of HIV testing location	Numbers (*n*)	4	0
		Percentage (%)	4.4%	0.0%
	Perceived healthy status	Numbers (*n*)	60	11
		Percentage (%)	65.9%	100.0%
	Stigma associated with receiving HIV care	Numbers (*n*)	19	0
		Percentage (%)	20.9%	0.0%

Barriers to HIV care (system)	Geographic barriers to healthcare facilities	Numbers (*n*)	0	0
		Percentage (%)	0.0%	0.0%
	Specialist shortages at primary care facilities	Numbers (*n*)	0	0
		Percentage (%)	0.0%	0.0%
	Incorrect clinical judgement leading to late/missed diagnosis	Numbers (*n*)	1	0
		Percentage (%)	1.1%	0.0%
	Lack of referral tracking from the initial care provider	Numbers (*n*)	4	2
		Percentage (%)	4.4%	18.2%
	Late referral to the treatment centre	Numbers (*n*)	4	0
		Percentage (%)	4.4%	0.0%
	No healthcare system barrier	Numbers (*n*)	82	9
		Percentage (%)	90.1%	81.8%

*Note:* LPs who experienced barriers in the healthcare system were labelled as adverse events.

## Data Availability

The data supporting this study's findings are available from the corresponding author upon reasonable request.

## Nomenclature

AIDS: Acquired immunodeficiency syndrome
ART: Antiretroviral therapy
CD4: Cluster of differentiation 4
HIV: Human immunodeficiency virus
ID: Infectious disease
IVDU: Intravenous drug user
LP: Late presenter
MSM: Men who have sex with men
NLP: Nonlate presenter
OI: Opportunistic infection
